# Low SOX2 expression marks a distinct subset of adenoid cystic carcinoma of the head and neck and is associated with an advanced tumor stage

**DOI:** 10.1371/journal.pone.0194989

**Published:** 2018-03-29

**Authors:** Julia Thierauf, Stephanie E. Weissinger, Johannes A. Veit, Annette Affolter, Natalia K. Laureano, Dirk Beutner, Gregor Heiduschka, Lorenz Kadletz, Moritz Meyer, Alexander Quaas, Peter Plinkert, Thomas K. Hoffmann, Jochen Hess

**Affiliations:** 1 Department of Otorhinolaryngology, Head and Neck Surgery, Heidelberg University Hospital, Heidelberg, Germany; 2 Department of Otorhinolaryngology, Head and Neck Surgery, University Medical Center Ulm, Ulm, Germany; 3 Institute of Pathology, University Medical Center Ulm, Ulm, Germany; 4 Institute of Pathology, Heidelberg University Hospital, Heidelberg, Germany; 5 Research Group Molecular Mechanisms of Head and Neck Tumors, German Cancer Research Center (DKFZ), Heidelberg, Germany; 6 Department of Oral Pathology, Federal University of Rio Grande do Sul, Porto Alegre, Brazil; 7 Department of Otorhinolaryngology, Head and Neck Surgery, University Hospital Cologne, Cologne, Germany; 8 Department of Otorhinolaryngology, Head and Neck Surgery, University Medical Center Göttingen, Göttingen, Germany; 9 Department of Otorhinolaryngology, Head and Neck Surgery, Vienna General Hospital, Vienna, Austria; 10 Institute of Pathology, University Hospital Cologne, Cologne, Germany; University of South Alabama Mitchell Cancer Institute, UNITED STATES

## Abstract

**Introduction:**

The transcription factor SOX2 has been identified as a lineage survival oncogene in squamous cell carcinoma and copy number gain is a common event in several human malignancies including head and neck cancer. However, the regulation and function of SOX2 during carcinogenesis as well as its prognostic value appears to be highly context dependent. As an example, high SOX2 expression in lung squamous cell carcinoma (SCC) is related to a favorable prognosis, while it is associated with poor outcome in lung adenocarcinoma. More recently, higher SOX2 levels and improved survival was also reported for head and neck SCC (HNSCC), and silencing of SOX2 expression in HNSCC cell lines revealed a mesenchymal-like phenotype with prominent vimentin expression. So far, SOX2 expression and its clinical relevance for other head and neck cancers, such as adenoid cystic carcinoma (HNACC) have not been sufficiently investigated.

**Material and methods:**

SOX2, vimentin and E-cadherin expression was assessed by immunohistochemical staining on serial sections from formalin fixed and paraffin embedded tissue samples of a patient cohort (n = 45) with primary ACC and correlated with patient and tumor characteristics as well as survival.

**Results:**

High SOX2 expression was found in 14 (31%) primary tumor specimens and was significantly correlated with a N0 lymph node status (p = 0.04), while low SOX2 expression was correlated with a solid growth pattern (p = 0.031). Of the 45 patients, 27 tumor samples resembled an EMT-like phenotype, as assessed by high vimentin and low E-cadherin levels. However, in HNACC SOX2 levels were neither correlated with vimentin nor with E-cadherin expression, further supporting a context dependent regulation and function of SOX2 in distinct tumor entities.

**Conclusion:**

The absence of SOX2 was predominantly found in solid HNACC, which are characterized by a more aggressive phenotype in ACC. However, the underlying molecular mechanisms of SOX2 regulation and function in distinct HNACC subgroups remain to be fully elucidated.

## Introduction

Recurrent gene amplification of the transcription factor SOX2 on human chromosome 3q is a common feature in the development of squamous cell carcinoma (SCC), but its function during carcinogenesis and prognostic value are highly context dependent [1]. While high SOX2 expression promotes lung SCC, adeno-like carcinoma develop largely in the absence of SOX2 [2]. Although lung adenocarcinoma (ADC) harbor significantly lower amounts of SOX2 as compared to SCC, SOX2 overexpression has mostly been correlated with a poor clinical outcome. In contrast, several studies reported a positive association between SOX2 expression and improved survival in lung SCC. High SOX2 expression was also correlated with better survival in other tumor entities despite the well-established function of SOX2 in tumor-relevant processes [3], [4], [5]. Bayo et al. provided a possible explanation by demonstrating that silencing of SOX2 expression in head and neck squamous cell carcinoma (HNSCC) cell lines with 3q amplification induces a mesenchymal-like phenotype with increased expression of well-established mesenchymal marker genes (e.g. VIM and FN1), which was accompanied by accelerated tumor cell motility and invasion [6]. An inverse regulation of SOX2 and mesenchymal marker genes in primary HNSCC is also evident from public available data of The Cancer Genome Atlas [7]. However, it is worth noting that expression of epithelial markers, such as E-cadherin were not affected by SOX2 silencing in HNSCC cell lines indicating a partial but not a complete epithelial-to-mesenchymal transition [6]. Moreover, the expression of SOX2 and its correlation to clinical outcome are highly variable among different tumor entities. However, the role of SOX2 in salivary gland malignancies like the adenoid cystic carcinoma (ACC) has not been sufficiently elucidated.

Patients with head and neck adenoid cystic carcinoma (HNACC) suffer from high rates of local tumor recurrences and distant metastasis predominantly of the lung. HNACCs are characterized by an infiltrative growth pattern along nerve tracks and a high rate of treatment failure. Although the incidence is low (1–2 per million per year [8]), HNACC is one of the most common cancer types of the salivary glands [9]. HNACC can be subdivided into 3 growth patterns, the cribriform, the tubular and the solid type, the latter subtype being the most aggressive one. Due to the small number of cases, our knowledge about the molecular mechanisms of tumor progression and the formation of metastasis in HNACC remains limited.

Because of the tenacious metastatic behavior of ACC, this study aims to elucidate the expression and clinical relevance of SOX2 in this rare but aggressive tumor entity with dismal survival rates for many patients.

## Material and methods

### Ethics

This study was approved by the Institutional Review Board of Ulm University Medical Center (approval number: 304/13) and was conducted in accordance with the Principles of the Declaration of Helsinki.

### Study population

Medical records of 45 patients with the primary diagnose of HNACC were collected retrospectively from the University Hospital Ulm, Germany, University Hospital Cologne, Germany and the University Medical Center Vienna, Austria. Paraffin-embedded tumor samples were available from all patients. All samples were diagnosed by a H&N specialized pathologist. The study was endorsed by the local ethics committees (Ethikkommission der Universität Ulm, approval number Ulm: 304/13, Ethikkommission der Universität Köln, Cologne: 17–412, Ethikkommission der Universität Wien, Vienna: 1926/2015), the institutional review boards and was conducted in accordance with the Principles of the Declaration of Helsinki. Clinical data included basic demographic data such as age, gender, date of diagnosis, time to recurrence, death and last follow-up as well as tumor characteristics like localization, tumor type, TNM and stage (Table 1). All patients received primary tumor resection with neck dissection, either modified-radical neck dissection or, in case of extensive nodal invasion, radical neck dissection. Progression was defined as tumor recurrence or progressive tumor growth in either local, loco-regional or distant site. Overall-survival (OS) was assessed from the time point of initial diagnose (date of pathologic report) to last follow-up or date of death. The recurrence-free survival (RFS) was assessed from initial treatment in number of month.

**Table 1:** clinical and pathological features of the ACC cohort according to SOX2 expression.

		SOX2^negativ^	SOX2^positiv^	p-value
**Age [years]**	≤55	13	8	0.344
	>55	18	6	
**Gender**	male	13	5	0.693
	female	18	9	
**Localization**	extra-parotideal	18	12	0.069
	parotideal	13	2	
**AJCC**	I, II III, IV	13 18	10 4	0.067
**T status**	1+2	11	8	0.173
	3+4	20	6	
**N status**	N0	20	13	**0.047**
	N+	11	1	
**M status**	0	30	13	0.555
	1	1	1	
**Progression**	no	20	9	0.988
	yes	11	5	
**Growth pattern**	cribrif./tubul./mix.	19	13	**0.031**
	solid	12	1	
**Localization of non-solid HNACC**	Extra-parotideal	1	4	**0.015**
	parotideal	7	1	

Significant p-values (<0.05) are indicated in bold

### Immunohistochemistry and co-immunofluorescence

For immunohistochemistry, 3 μm thick sections were cut from formalin fixed and paraffin embedded tumor tissue and stained according to the manufacturer’s instructions. Heat-induced antigen retrieval was carried out by steaming sections (on Superfrost Plus, Menzel, Braunschweig, Germany) for 30 minutes in 10 mM citrate buffer pH6 (Multi Gourmet Steamer, Braun, Germany). Immunohistochemistry was conducted with the Impress HRP Reagent Kit (Vector Laboratories, USA) using following antibodies: anti-E-cadherin antibody (Santa Cruz Biotechnology, USA, sc7870 polyclonal rabbit), anti-vimentin antibody (Progen, Germany, VIM 3B4, monoclonal mouse), anti-SOX2 antibody (Cell Signaling, USA, D609 rabbit mAb), anti-ki67-antibody (Dako, Germany, MIB-1 monoclonal mouse), and anti-pan-Cytokeratin-antibody (Progen, Germany, polyclonal guinea pig). To visualize staining an AEC-System (Peroxidase substrate kit AEC SK-4200, Vector Laboratories, USA) or a DAB-System (Peroxidase substrate kit DAB SK-4100, Vector Laboratories, USA, respectively) were used. Counterstaining was done with hematoxylin-eosin solution modified according to Gill III (Merck, Germany) to visualize tissue integrity. For immunofluorescence staining, secondary antibodies labeled with either an Alexa Fluor^®^ 488 or Cy^®^3 dye (ThermoFisher, Germany) were used for visualization, as well as Hoechst 33342 Solution (ThermoFisher, Germany). Ki67 and SOX2 staining was nuclear, whereas staining for the other markers were membranous, as better visualized in the IF pictures. As an internal control, adjacent normal tissue was used. Nonspecific binding was used by the application of secondary antibodies solely for negative controls as well as Isotype Controls (S1 Fig).

Stained sections were evaluated according to the relative amount of positive tumor cells (1 = 0% positive cells, 2 = less than 33%, 3 = between 34% and 66%, 4 = more than 66% positive tumor cells) [6]. The score was used by three independent observers to evaluate the final staining results. Negative controls omitting the application of primary antibodies were used in every assay. The histological subtype of ACC was defined as the predominant growth pattern in one tissue section. Patient subgroups were arranged according to vimentin^high^ (score 3–4) or vimetin^low^ (score 1–2) expression and to E-cadherin^high^ (score 3–4) or E-cadherin^low^ (score 1–2) expression. Any staining of SOX2 in the nucleus was scored as positive.

### Statistical analyses

The expression of vimentin, E-cadherin and SOX2 were correlated to clinical characteristics and survival times of each patient. Statistical analysis was done using SPSS (version 21, IBM, USA) statistics software. Differences between groups were assessed using the Chi square test. Survival times of patients who were alive were censored. PFS was calculated from the initial tumor-therapy until the date of the first local recurrence, lymph node or distant metastasis, second primary carcinoma, or date of cancer-related death within the follow-up period (events). Patients without progression (no event) or cancer-unrelated death were censored. Kaplan–Meier graphs were blotted to estimate survival distributions, and statistical significance between groups was calculated by log-rank tests. In all statistical tests, a p-value of 0.05 or below was considered as statistically significant.

## Results

**Fig 1:**
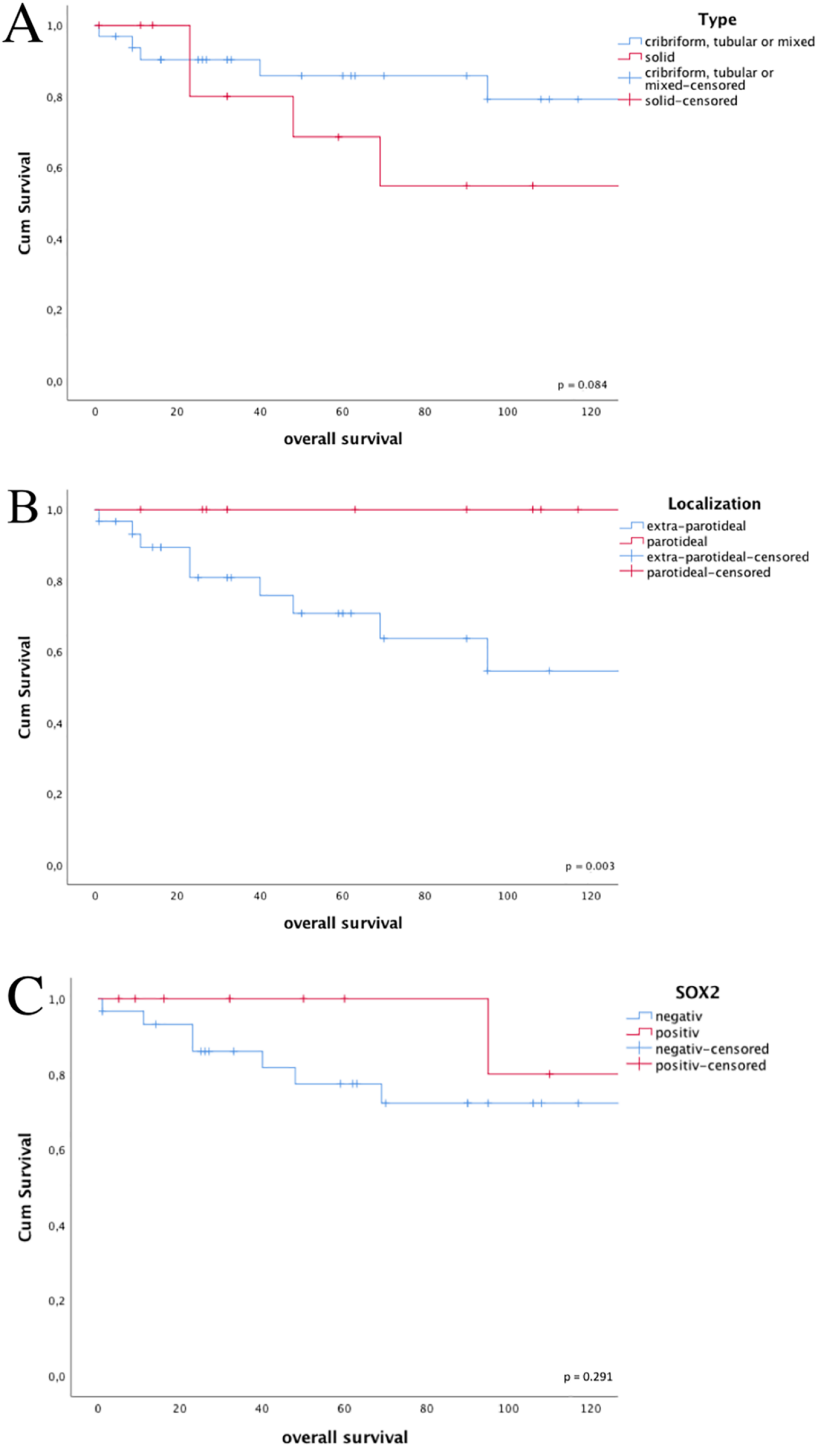
A) solid growth patterns of ACC show a trend towards a worse clinical outcome as assessed by Kaplan-Meier survival analysis, B) extra-parotideal ACCs show significantly worse overall survival C) SOX2 positive ACCs show prolonged overall survival compared to tumors with a loss of SOX2. M+ positive were excluded. Fig 1A post-hoc power: 30.7%, Fig 1B post-hoc power: 49.9%, Fig 1C post-hoc power: 15.8%.

The cohort of 45 patients showed a mean age of 55 years and 60% were females (Table 1). 33% of HNACC cases were found in the parotideal gland and 29% showed a solid growth pattern. Most of the cases were either stage I or stage IVA at initial diagnosis and 36% of patients showed tumor progression during the follow-up period. Patients diagnosed with a solid tumor type showed a trend towards poor clinical outcome (Fig 1A, p = 0.084) and that parotideal HNACC had a better overall survival (Fig 1B, p = 0.003), which is in line with findings of other studies [8], [9].

### SOX2 expression correlates with nodal status and growth pattern of HNACC

**Fig 2:**
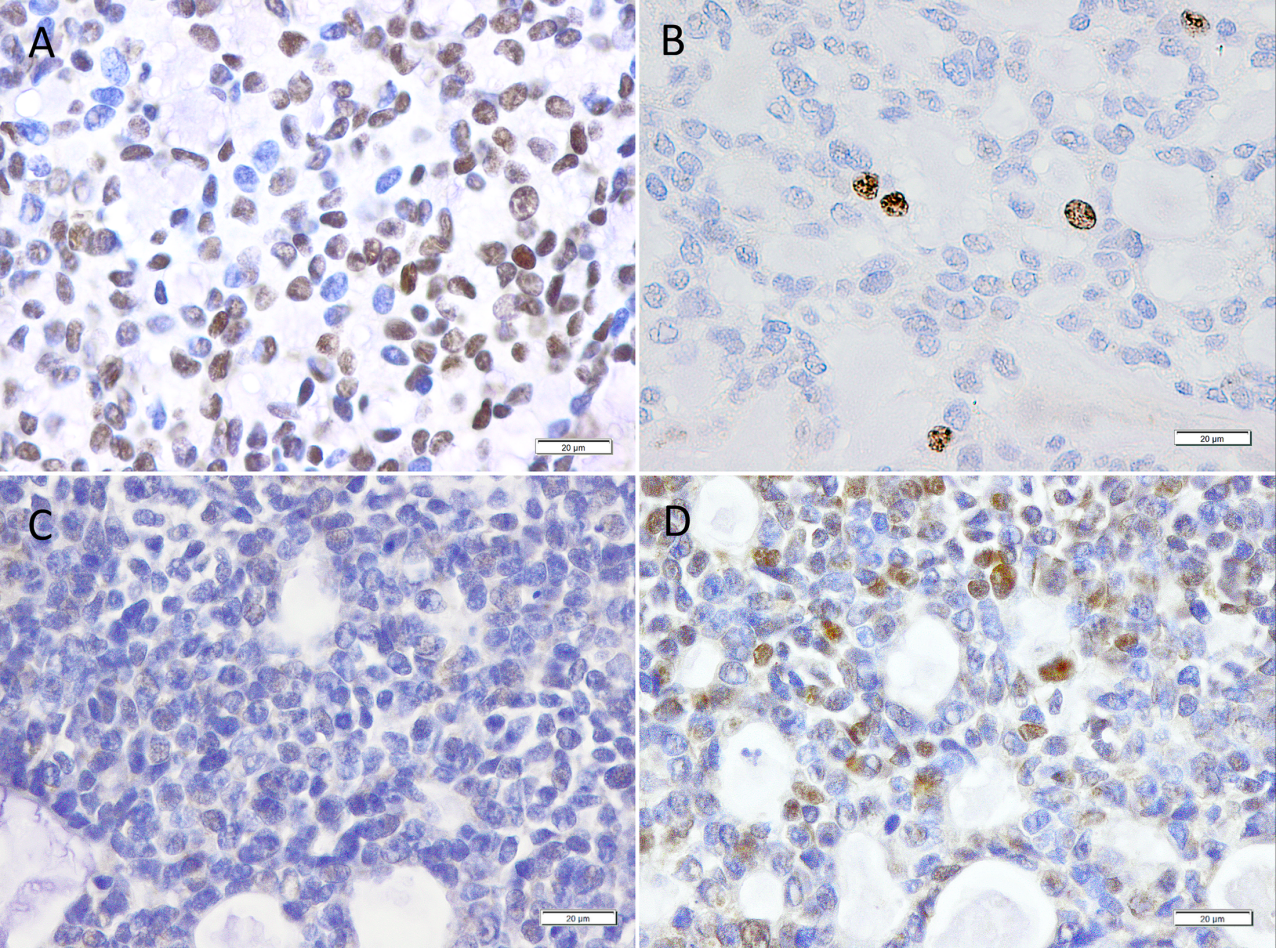
Representative microscopic images of tumor samples from 2 patients with an ACC. A) shows a tumor sample with a high SOX2 expression using immunohistochemistry (in 4x and 40x magnification) and B) an almost absent expression of ki67. C) shows a tumor sample with no detectable SOX2 expression and D) with elevated levels of ki67.

In total, 14 out of 45 (31%) HNACC revealed a prominent nuclear staining for SOX2 (Fig 2). Overall, SOX2 expression was not significantly correlated with gender, age or localization of the tumor (Table 1). Interestingly, SOX2 expression was significantly higher in patients without lymph node metastasis as compared to their N+ counterparts (p = 0.047), and a clear trend of higher SOX2 expression in patients with a lower tumor stage (p = 0.067) was observed. Moreover, solid types of HNACCs were characterized by low SOX2 expression (p = 0.031). In non-solid HNACC, SOX2 was detected preferentially in the extra-parotideal tumors, whereas the parotideal tumors did not show relevant SOX2 expression (p = 0.015). To address the question, whether SOX2 serves as prognostic biomarker for clinical outcome, we performed Kaplan-Meier blots for overall survival (OS) of patients (Fig 1). SOX2 expression was not associated with prolonged OS as compared to HNACCs lacking detectable SOX2 levels. Since tumors with a higher tumor stage seemed to undergo a loss of SOX2 (Table 1), we performed immunohistochemical analyses to measure proliferation with an anti-ki67-antibody in representative tumor samples with high, low and absent expression of SOX2. The tumor with the highest SOX2 expression showed almost no ki67 expression. Nuclear Ki67 expression in tumor cells was increased in SOX2 negative samples. The inverse correlation of ki67 and SOX2 is demonstrated in Fig 2, Isotype control is provided in S1 Fig.

### SOX2, vimentin and E-cadherin expression in HNACC

Since an inverse regulation of SOX2 and expression of mesenchymal markers has been postulated in primary HNSCC, the expression of well-established EMT-markers such as vimentin and E-cadherin was analyzed in our ACC cohort. In total, 27 (60%) tumor samples revealed a high vimentin but low E-cadherin expression pattern, while 10 (22.2%) showed a co-expression of both proteins (S2 Fig).

**Fig 3:**
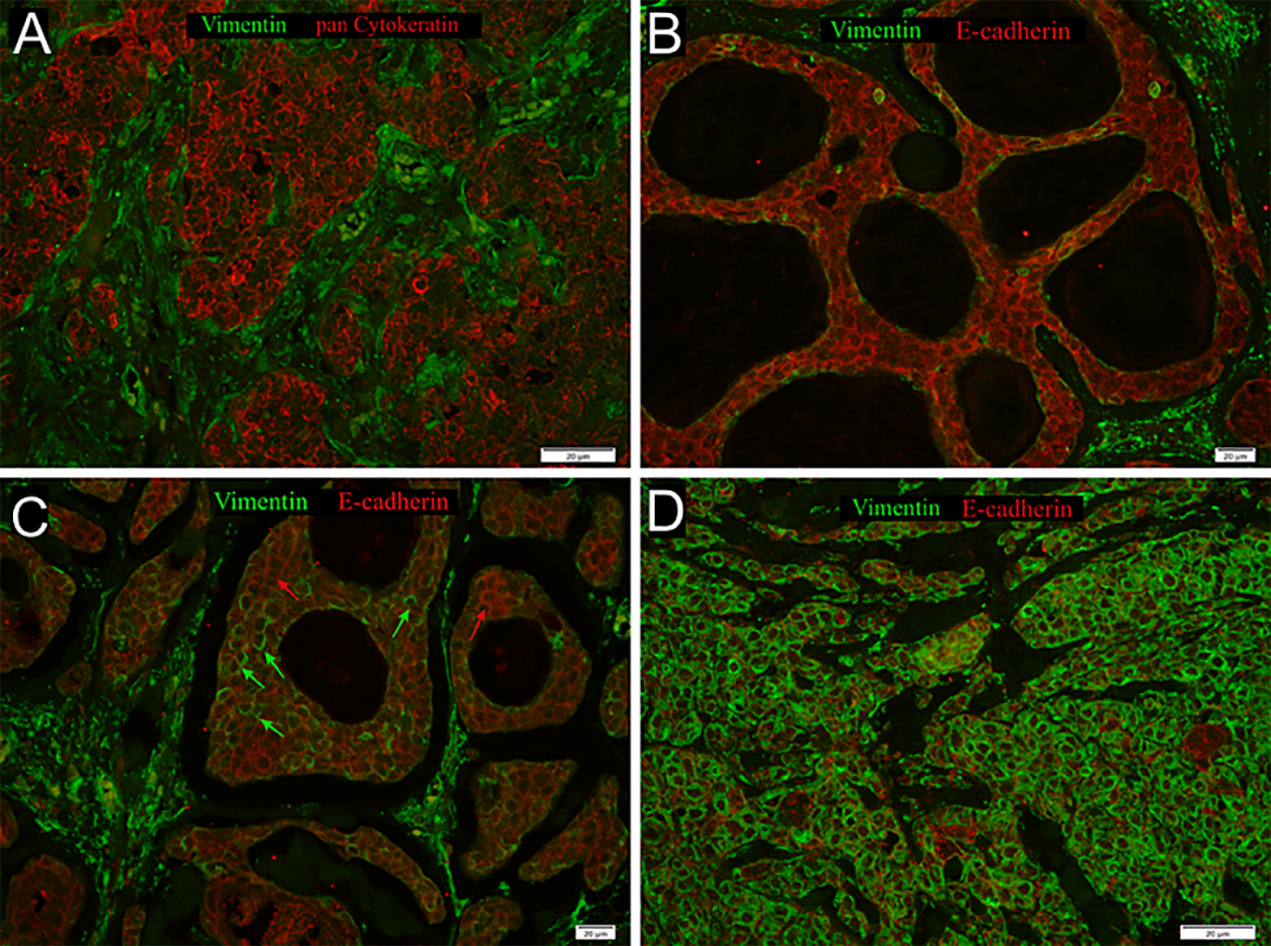
Co-Immunofluorescence staining with anti-vimentin (green) and anti-pan cytokeratin (red) of an ACC sample (A) with no overexpression of vimentin, B) Co-Immunofluorescence staining with anti-vimentin (green) and anti-e-cadherin (red) of an ACC sample without vimentin expression but e-cadherin overexpression, C) with moderate vimentin overexpression marked by green and detectable e-cadherin expression marked by red arrows, D) high vimentin expression in almost all tumor cells.

Since vimentin-positive tumor cells are rather difficult to distinguish from stromal cells by IHC staining, especially in the solid type of ACCs, we performed co-immunofluorescence (co-IF) staining using an anti-vimentin antibody together with an anti-pan-cytokeratin antibody (Fig 3). In total, 26 out of 31 SOX2^negtive^ tumors showed high expression of vimentin in tumor cells. However, the correlation between SOX2, vimentin or E-cadherin did not reach statistical significance (S1 Table). Moreover, vimentin and E-cadherin protein expression did not correlate with survival or any clinical or histo-pathological feature tested, including age, TNM status, clinical stage and pathological grade (data not shown).

## Discussion

In the past, SOX2 was identified as a novel lineage survival oncogene in lung and esophageal SCC [1], and numerous experimental data support the assumption that SOX2 promotes cell motility, proliferation and invasive capacity of cancer cells [10], [11], [12], [13]. However, recent studies support a more complex model in which the regulation and function of SOX2 during neoplastic transformation and malignant progression is highly context dependent and critically involved in tumor cell plasticity. In line with this assumption, the prognostic value of SOX2 expression differs between distinct tumor entities [10]. Ferone et al. provided experimental evidence for the context-dependent function of SOX2 during carcinogenesis. In genetically-modified mouse models of lung cancer they confirmed that SOX2 is a key oncogenic driver in the development of lung SCC [2]. In contrast, loss of SOX2 induced more adeno-like tumors demonstrating that SOX2 acts as a determining oncogenic switch in promoting lung SCC from different cells of origin.

Dai et al. evaluated the expression of SOX2 in ACC tissue using IHC, western blot, and qPCR analysis [14]. They concluded that SOX2 expression was associated with the development of ACC and that SOX2 expression was significantly correlated with advanced T stage and distant metastasis. Moreover, the univariate and multivariate analyses clearly demonstrated that SOX2 expression was a statistically significant risk factor affecting OS and DFS of patients with ACC. However, the group does not provide data concerning the nodal status of the patients.

In our cohort, SOX2 was detected in 14 out of 45 (31%) samples and was significantly higher in patients without lymph node metastasis at initial diagnosis; only one patient with a SOX2^positive^ tumor showed positive lymph nodes. In total, a reduced risk of lymph node metastasis for tumors with high SOX2 expression was reported already for esophageal and oral cancer by Chuang et al and Züllig et al [15], [16]. Dai et al. do not give information on lymph node metastases of their cohort, but describe a high rate of distant metastases (67%), whereas the M1 status of our cohort is 4% (2/45). Moreover, 59% of their cohort showed a low T stage (T1 and T2) whereas in our cohort, the higher tumor stages were predominant (T3 and T4, 58%). The contradictory findings in the cohort of Dai et al.—who describe SOX2 as an unfavorable risk factor for ACCs—and our cohort might be explained by those differences within the two ACC cohorts.

Interestingly, in our ACC cohort, only one patient with a solid tumor type was SOX2 positive. The solid growth pattern of ACC is known to be the most aggressive type of ACC with the most unfavorable clinical outcome [8]. The absence of SOX2 in solid ACCs might explain at least partially their poor outcome. Additionally, only four SOX2^positive^ patients had stage III or IV ACCs. Toschi et al. observed significant better survival rates of SOX2 positive lung cancer patients, especially in early stage. They showed, that increased SOX2 and FGFR1 gene copy number is a common event in squamous cell lung cancer patients and could demonstrate their improved overall survival [15]. In our ACC cohort, tumors with the highest T and AJCC stage showed the lowest levels of SOX2. In representative tumor samples with high, low and absent SOX2 expression, SOX2 was negatively correlated with ki67. Studies describing SOX2 as a negative predictive factor for survival in other malignancies, observed a simultaneous co-expression of SOX2 and ki67, indicating their positive correlation [17]. However, Otsubo et al. [5] could demonstrate, that exogenous SOX2 inhibits proliferation of gastric epithelial cell lines, that SOX2 plays a crucial role in gastric carcinogenesis as a tumor suppressor and that loss of SOX2 expression may cause gastric epithelial cells develop into carcinomas. Overall, these different publications underline the hypothesis of a context dependent role of SOX2.

In line with previous findings on HNSCC [6], 24 out of 31 SOX2^negtive^ tumors showed high expression of vimentin in tumor cells, indicating a more mesenchymal phenotype. However, the correlation between SOX2 and vimentin expression did not reach statistical significance. Moreover, vimentin or E-cadherin protein expression did not correlate with survival or any clinical or histo-pathological feature tested. Nevertheless, Siu et al. [18] showed in ACCs, that the solid form expresses αvβ6 integrin and tenascin-C, which are known promoters of EMT. They also observed high levels of E-cadherin in an ACC cell line with the characteristics of the cribriform type and high levels of vimentin and of αvβ6 integrin in the more myoepithelial-like cell line.

## Conclusion

Our work underlines the difference between the cribriform/tubular and the solid growth pattern by significantly lower SOX2 levels. We showed that SOX2 up-regulation is associated with decreased risk of lymphatic metastasis in HNACC. Moreover, the loss of SOX2 was predominantly observed in solid ACCs as well as advanced tumor stages. Therefore, we could indicate, that SOX2 might act as a determining oncogenic switch in promoting several ACC growth patterns from different cells of origin. Taken together, our results argue for larger cohorts of ACCs and detailed information of different subgroups. Future studies to (i) foster this hypothesis and to (ii) unravel relevant SOX2-related signaling and gene regulatory networks in ACC, will be required.

## Supporting information

S1 Fig Microscopic image of our Test-TMA with Isotype control for SOX2 using Cell Signal rabbit (DA1E) mAb IgG XP^®^Isotype control. (TIFF)

S2 Fig Representative microscopic images of a negative vimentin staining in an ACC tumor sample (A): 4x magnification, B): 20x magnification). Pictures C) and D) show the simultaneous overexpression of e-cadherin in the same sample (4x and 20x). E) Representative microscopic images of a EMT-phenotype ACC, assed by a vimentin overexpressing tumor sample in 4x magnification and 20x magnification (F). Pictures G) and H) show the absence of e-cadherin in the same sample (4x and 20x).
(TIFF)

S1 Table Distribution of EMT-markers in SOX2 positive and negative ACCs. (DOCX)
