# Supplementary figures and images for: Low SOX2 expression marks a distinct subset of adenoid cystic carcinoma of the head and neck and is associated with an advanced tumor stage
Source: PLoS One. 2018 Mar 29;13(3):e0194989. doi: 10.1371/journal.pone.0194989 (PMC5875788; doi:10.1371/journal.pone.0194989)

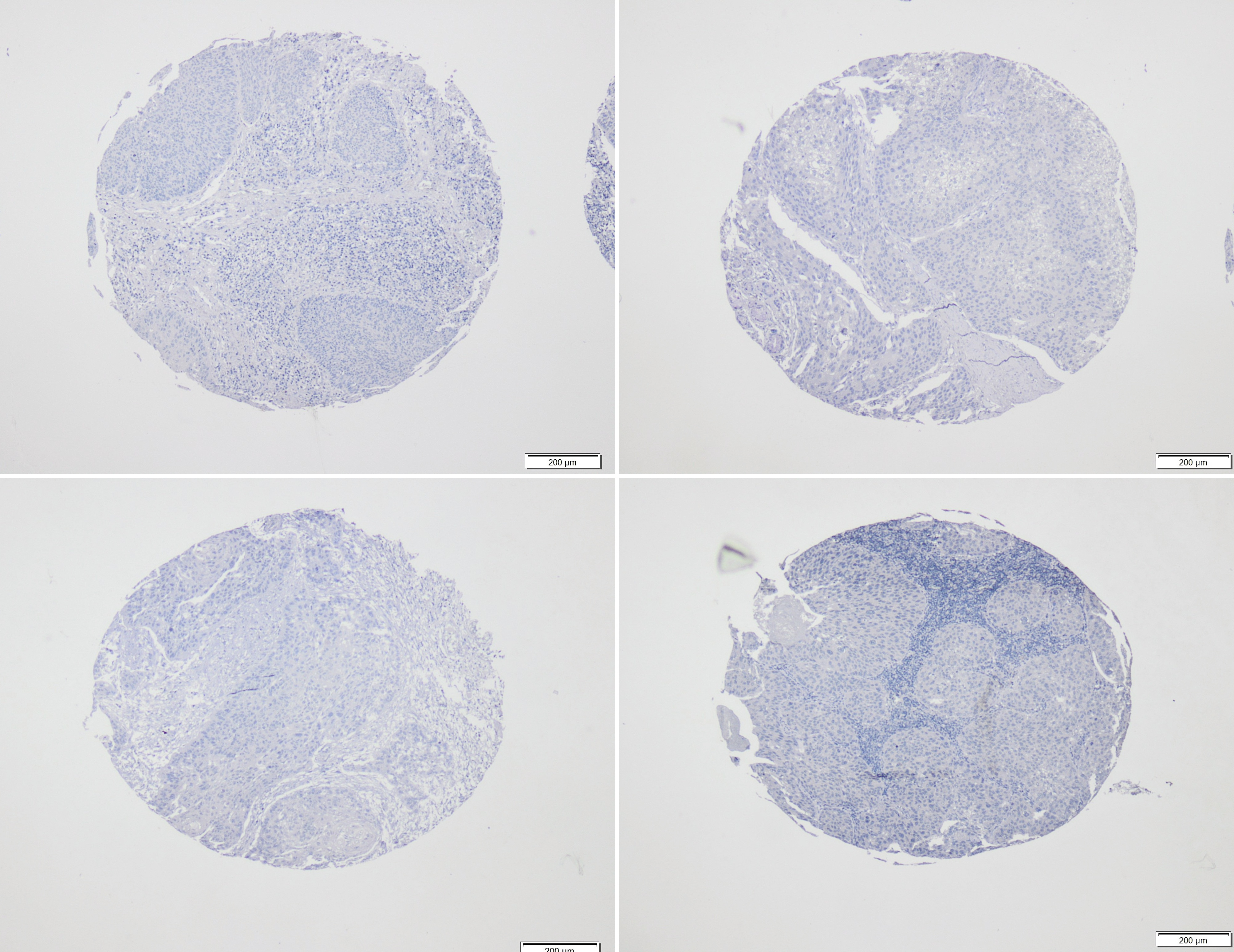

Supplement: S1 Fig — (TIFF) [file pone.0194989.s001.tiff]

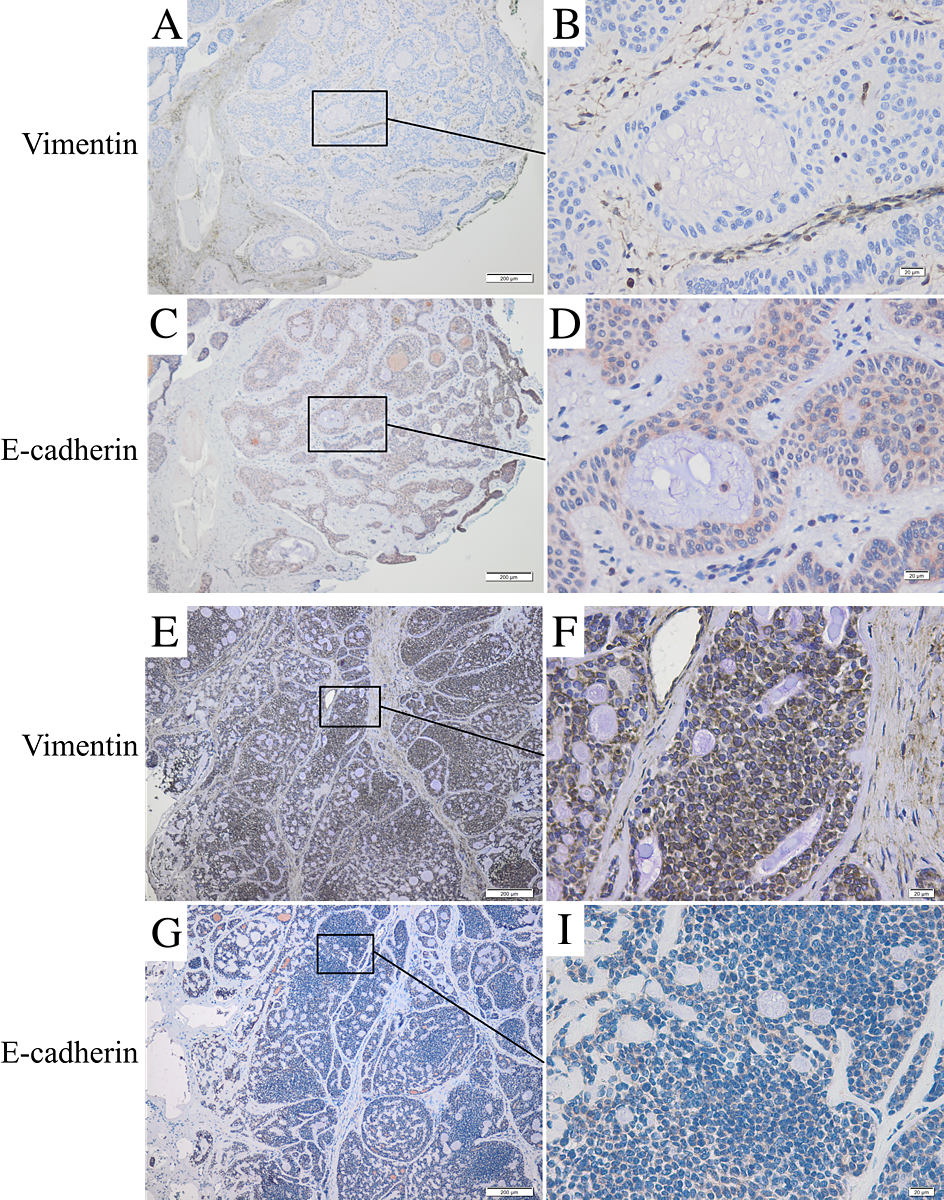

Supplement: S2 Fig — Representative microscopic images of a negative vimentin staining in an ACC tumor sample (A): 4x magnification, B): 20x magnification). Pictures C) and D) show the simultaneous overexpression of e-cadherin in the same sample (4x and 20x). E) Representative microscopic images of a EMT-phenotype ACC, assed by a vimentin overexpressing tumor sample in 4x magnification and 20x magnification (F). Pictures G) and H) show the absence of e-cadherin in the same sample (4x and 20x). (TIFF) [file pone.0194989.s002.tiff]
