# Supplementary material for: Low SOX2 expression marks a distinct subset of adenoid cystic carcinoma of the head and neck and is associated with an advanced tumor stage
Source: PLoS One. 2018 Mar 29;13(3):e0194989. doi: 10.1371/journal.pone.0194989 (PMC5875788; doi:10.1371/journal.pone.0194989)
Supplement: S1 Table — (DOCX) [file pone.0194989.s003.docx]

|  | | **SOX2** | | ***p*** | **E-Cadherin** | | ***p*** |
| --- | --- | --- | --- | --- | --- | --- | --- |
|  |  | negative | positive |  | negative | positive |  |
| **Vimentin** | negative | 5 | 0 | 0.111 | 2 | 3 | 0.598 |
|  | positive | 26 | 14 |  | 21 | 19 |  |
| **E-Cadherin** | negative | 13 | 10 | 0.067 |  |  |  |
|  | positive | 18 | 4 |  |  |  |  |

Table S1: Distribution of EMT-markers in SOX2 positive and negative ACCs
